# Influence of Conventional Polymer, Hybrid Polymer and Zinc Phosphate Luting Agents on the Bond Strength of Customized Zirconia Post in Premolars—An In-Vitro Evaluation

**DOI:** 10.3390/polym14040758

**Published:** 2022-02-15

**Authors:** Khulud A. AlAali, Abdulaziz AlHelal, Jawaher R. Almahri, Aroob A. Albakri, Ragad M. Albani, Yasmeen A. Alhaizan, Mai M. Alhamdan, Naif A. Alaql, Mashael Binhasan, Eman M. Alhamdan, Ahmed H. Albaqawi, Fahim Vohra, Tariq Abduljabbar

**Affiliations:** 1 Department of Clinical Dental Sciences, College of Dentistry, Princess Nourah Bint Abdulrahman University, Riyadh 11671, Saudi Arabia; 2 Prosthetic Dental Science Department, College of Dentistry, King Saud University, Riyadh 11545, Saudi Arabia; alhelal.abdulaziz@gmail.com (A.A.); mayalhamdan@ksu.edu.sa (M.M.A.); ealhamdan@ksu.edu.sa (E.M.A.); fvohra@ksu.edu.sa (F.V.); 3 Prosthodontics Postgraduate Resident, College of Dentistry, Princess Nourah Bint Abdulrahman University, Riyadh 11671, Saudi Arabia; jawaherrm@gmail.com; 4 General Dentist, College of Dentistry, Princess Nourah Bint Abdulrahman University, Riyadh 11671, Saudi Arabia; aroob.ahmed.b@gmail.com; 5 Prosthodontics Postgraduate Resident, College of Dentistry, King Saud University, Riyadh 11671, Saudi Arabia; ragad.albani@gmail.com; 6 Restorative Postgraduate Resident, King Faisal Specialist Hospital, Riyadh 11211, Saudi Arabia; yasmeenalhaizan@gmail.com; 7 Prosthodontic Department, Riyadh Specialized Dental Center, Ministry of Health, Riyadh 13226, Saudi Arabia; drnaifalaql@hotmail.com; 8 Department of Restorative Dentistry, Division of Operative Dentistry, College of Dentistry, King Saud University, Riyadh 11545, Saudi Arabia; mbinhasan@ksu.edu.sa; 9 Department of Restorative Dental Science, College of Dentistry, University of Ha’il, Ha’il 55476, Saudi Arabia; a.albaqawi@uoh.edu.sa

**Keywords:** adhesion, bond strength, resin polymer, glass ionomer, zinc phosphate

## Abstract

The aim was to identify the influence of conventional polymeric resin based cement (RC), hybrid polymer modified glass ionomer (RMGIC) and Zinc phosphate cement (ZPC) on the pull out strength of the customized zirconia post in premolars. Access cavity and root canals were performed in sixty premolar teeth with the standardized crown down technique (ProTaper Universal, Dentsply). Post space impressions were scanned, and the pre-sintered Zenostar Zr Translucent blanks (Weiland Dental, Pforzheim) were milled with the Opera-system to form the post. All prepared specimens were divided equally in three groups based on the cement type employed for luting as follows: group A: ZPC; group B (GC Fuji PLUS Capsule): RMGIC; group C (and RC (3M RelyX ARC). Ten specimens in each group were thermocycled (TC) at 5 and 55 °C in distilled water baths (40,000 cycles). Pull out bond strength was assessed using a universal testing machine at 0.5 mm/min. The means and standard deviations were compared using ANOVA and Tukey Kramer multiple comparisons tests. A significant difference among the cement groups as well as between TC and non-thermocycled (NTC) groups (*p* < 0.05) was observed. The highest tensile stress was demonstrated among group C (Resin, 69.89 ± 4.81 (NTC), 64.06 ± 4.36 (TC)) with the least in group A, (zinc phosphate, 43.66 ± 5.02 (NTC), 37.70 ± 5.10 (TC)) for both groups. Group A presented with 100% adhesive bond failures, followed by 80% in group C and 70% in group B, respectively. A similar outcome was observed in the TC group for the cement; however, unlike the NTC group, the TC group showed more cohesive failures compared to the NTC mixed failure. Dual cure polymer based cement demonstrated higher bond strength and efficient adhesive bonding of the customized Zr post with root dentine compared to zinc phosphate (non-polymeric) and RMGIC (hybrid polymer). Thermocycling compromised Zr post adhesive bonding to root dentin.

## 1. Introduction

Posts and cores are essential parts of an endodontically restored tooth to strengthen the cervical tooth structure. In the past, cast metal posts were commonly used to support the remaining tooth structure of endodontically treated teeth [1]. Using a post in a damaged tooth withholds certain advantages, which include mechanical retention of the core and greater adaptation to customized root canal preparation; however, metal posts have a higher elastic modulus than the tooth structure, which may lead to root fracture [2]. Currently, the commonly employed post system is glass fiber, due to its better esthetics, adhesive bonding, translucency and ability to increase the fracture strength of the tooth [3].

The difference in the root canal structure is an important factor influencing the success of the post core system [4]. Morphological differences along the root canal often cause a mismatch of the post with respect to the diameter and post space, which increases the chance for thick cementation [4]. Preformed posts often present difficulty in adjusting to prepared canals and impose a risk of root fracture. Over the past few decades, a customized post and core were recognized as a standard method for superior mechanical properties as well as excellent biocompatibility for stable post and core treatment [5]. However, use of cast metal posts is discouraged due to grayish-blue discoloration of the teeth, difficult temporization, operator dependency and laboratory cost.

Zirconia oxide (ZrO) posts offer great potential for restoration of endodontically treated teeth due to high strength, increased cement polymerization, translucency and better fit of the customized post. Studies show that a zirconia post lined with composite polymer resin reduces the cement layer thickness, enhances frictional retention and creates a better adaptation to the root canal [6,7]. However, early failure was observed among posts as a result of the poor bonding to root dentin [7]. It is reported that cementation of the zirconia post with polymeric cement can be compromised due to incomplete cement polymerization, resulting in a reduced depth of resin tags [8]. Surface treatment including use of the laser shows promise to improve the adhesive bonding of zirconia to dentin with cements [9]. In addition, use of calcium flouride nano-particle incorporated dentin adhesives shows potential in improving the bond strength of the zirconia post to dentin [10]. Therefore, it is suggested that self adhesive-luting agents in the form of hybrid polymers can be employed for post cementation as they offer a greater degree of polymerization compared to conventional polymer based cements [11].

Different cements are applied for post cementation based on the mechanism of action, including Zinc phosphate cement (ZPC), resin modified hybrid glass ionomer cements (RMGIC) and resin cements (RC). Zinc phosphate cement uses an acid base setting reaction and provides frictional retention; in comparison, resin cements show a micromechanical adhesion of interlocking resinous material in conditioned tooth structure. However, the hybrid RMGIC shows a chemical bond of carboxyl groups to Ca ions of tooth and also a micromechanical aspect of retention to the tooth surface structure [12]. To optimize outcomes for post core procedures, dentists have explored different techniques and cementing protocols in association with conventional polymerizing resin to enhance post retention [13]. Although customized posts compared to preformed posts have shown better retention over a period, customized zirconia posts at many instances demonstrated early debonding [14,15]. Therefore, it is critical to assess the influence of different luting agents on the adhesive bond of a customized Zr post to root dentin. Hence, the purpose of this in vitro study is to evaluate the tensile bond strength of a customized Zr post to single rooted premolar teeth using conventional polymers, hybrid polymers and non-polymeric cements.

## 2. Materials and Methods 

### 2.1. Ethical Consideration

The study protocol was approved by the ethics review committee for using extracted patient teeth for experiments. The study was performed within the guidelines of the Helsinki declaration (1964) and its subsequent modifications. A laboratory in vitro study was conducted to evaluate the pull out bond strength of customized zirconia posts cemented with different luting agents in endodontically treated teeth.

### 2.2. Specimen Preparation 

Sixty intact single rooted maxillary and mandibular premolars extracted for orthodontic treatment with a minimum root length of 14 mm were collected. The specimens were stored in a solution of 0.9% saline at room temperature. Specimens were selected based on the criteria that they were non-carious, not treated endodontically and free of restorations. Teeth with multiple roots, curved roots and root canal abnormalities such as calcification and internal root resorption were excluded. 

The coronal part of the teeth was removed with a water-cooled diamond saw (Isomet 5000 Linear Precision Saw, Buehler Ltd., Lake Bluff, IL, USA). For root canal preparation, the access cavity was prepared for all thirty teeth divided into three groups randomly (*n* = 10). Instrumentation was performed with a crown down technique with rotary files (ProTaper Universal, Dentsply, Bellaigues, Switzerland). The apical preparation was kept at file 20, and the preparation was completed using ProTaper Universal Rotary Files SX-S2, F1 and F2 at 0.25mm. The canals were irrigated with 2 mL of 2.5% sodium hypochlorite (NaOCl) and dried with paper points throughout the preparation. A K-file (#35, Dentsply, Bellaigues, Switzerland) was used as a master file, and canals were irrigated with distilled water and obturated with gutta-percha (GP) (Dentsply, Bellaigues, Switzerland) by the lateral condensation technique. Canals were sealed with a canal sealer, AH Plus (Dentsply, Konstanz, Germany), and specimens were stored at 37 °C for 24 h.

**Figure 1:**
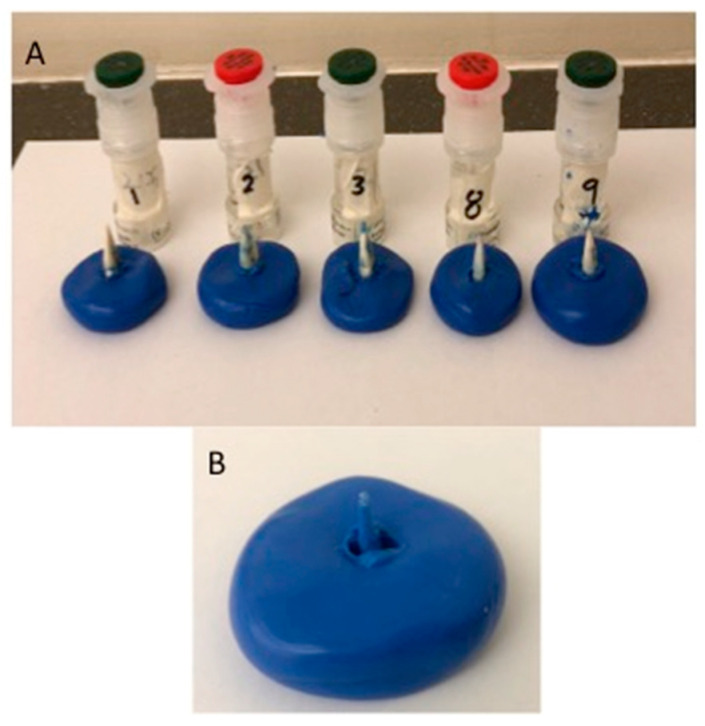
Indirect impressions (**B**) of study specimen (**A**).

**Figure 2:**
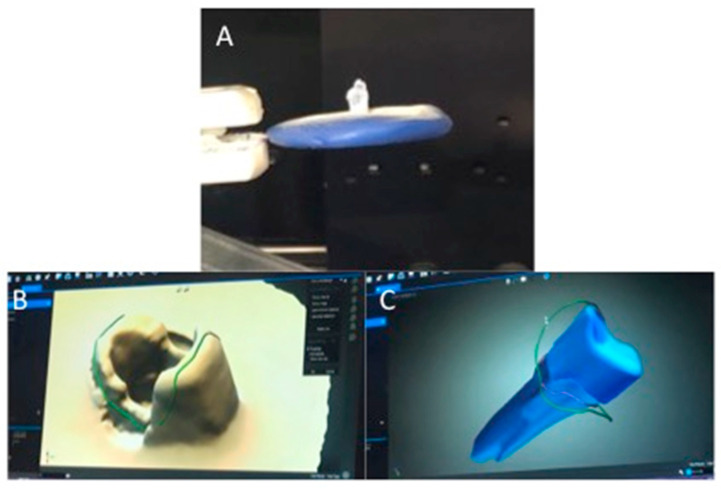
Post space impression scanning (**A**) and milling (**B**,**C**) of ZrO post using Opera System.

Initial preparation was performed, and post space preparation of all teeth was initiated by Peeso reamer No. 3-drill to a depth of 10–12 mm, keeping 3 mm at the apex to maintain the apical seal, followed by the use of a size one fiber post drill (3 M RelyX Fiber Post Drill). What was also used were 2 mL of NaOCl to irrigate root canals, which were later neutralized using 5 mL of distilled water. Teeth were assessed radiographically to ensure efficient post space. For Zirconia oxide (ZrO) post fabrication, an indirect technique was used to receive the zirconia post and core. SPEE-DEE plastic pins (Pulpdent, Watertown, MA, USA) were utilized with a rough surface. Thereafter, the impression was taken with the Virtual Putty Regular Set Refill Pack with the Virtual Heavy Body regular set (Ivoclar Vivadent-GmbH, Ellwangen, Germany) (Figure 1). Post space impressions were scanned, and the pre-sintered Zenostar Zr Translucent blanks (Weiland Dental, Pforzheim, Germany) were milled with the Opera-system according to the manufactures’ instructions (Figure 2A,B).

Prior to cementing the zirconia posts, teeth were divided into three groups of 20 specimens each. Specimens in group A were cemented using Zinc phosphate cement (non-polymeric cement) (ZPC) (DeTrey Zinc Phosphate cement, Dentsply Sirona, Charlotte, NC, USA). In group B, Zr posts were cemented using hybrid polymer resin modified glass ionomer cement (RMGIC) (GC Fuji PLUS Capsule, gc aMERICA, Alsip, IL, USA), and in group C, posts were cemented using polymeric resin-based cement (RC) (RelyX ARC, 3M ESPE, MN, USA). Surface treatment conditioning included silane application to all posts and the application of self-etching and an auto-polymerizing dentin primer (Ivoclar Vivadent, GmbH, Ellwangen, Germany) to root dentin. The protocol for cementation is presented in Table 1. From the twenty bonded teeth in each group, ten teeth were aged using thermocycling (TC) at 5 and 55 °C in distilled water baths (THE-1100, SD Mechatronik GmbH, Feldkirchen-Westerham, Germany). A total of 40,000 cycles for 30 s with a dwell time of 5 s were used for aging. The remaining bonded teeth in each group were not exposed to thermocycling and were kept safe in distilled water for one day.

### 2.3. Specimen Testing 

**Figure 3:**
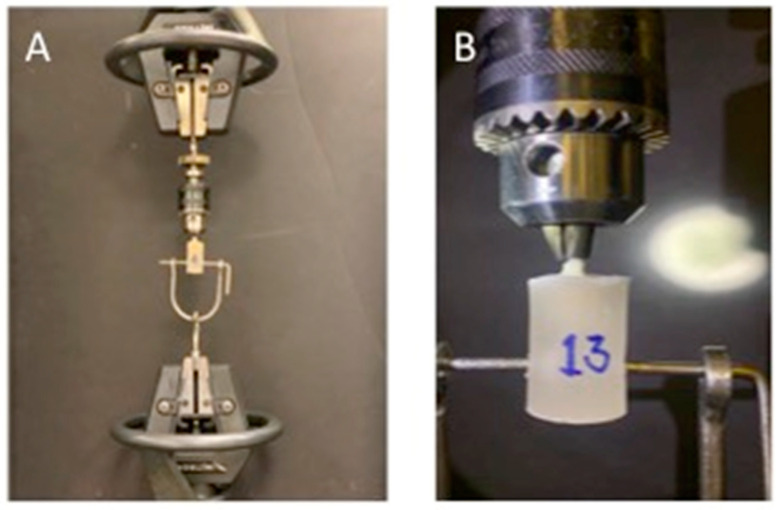
Pull out strength testing assembly using Universal Testing Machine (**A**) and magnified view (**B**).

Prior to tensile bond strength testing using the Universal Testing Machine (Instron, Norwood, MA, USA), it was ensured that all posts were 3 mm exposed from the preparation to facilitate a grip on posts. In addition, all teeth specimens were molded and inserted in acrylic resin (Techno Sin Resin—Protechno, Luxembourg, Belgium) to be in the long axis to avoid fracture (Figure 3). The pull out strength was determined by applying a control standard force using a direct tensile test at a ramp rate of 0.5 mm/min. The machine was computerized to calculate the tensile strength from the applied load, and the maximum load that caused specimen loss or fracture was recorded. The force applied was recorded in newtons (N).

### 2.4. Statistical Analysis

Normality of the data was obtained using the Kolmogorov–Smirnov test. The means and standard deviations of maximum load and tensile bod strength were compared using ANOVA and Tukey Kramer multiple comparisons tests. A p-value of <0.05 was considered statistically significant.

## 3. Results 

The present study evaluated the pull out strength of the zirconia post using zinc phosphate (non-polymeric), RMGIC (hybrid polymer) and RelyXarc (conventional polymer) luting cements. A significant difference in tensile bond strength among the cement groups was observed (*p* < 0.05). In addition, a significant difference in the tensile bond strength was observed between the TC and non-thermocycled (NTC) specimens in the respective cement groups (*p* < 0.05).

The highest mean value for maximum load at failure in both TC and NTC groups was in group C ((Relyx ARC: 98.77 ± 8.50 (TC), 123.51 ± 9.81 (NTC)), whereas the lowest means observed were in group A specimens (zinc phosphate: 43.20 ± 3.11 (TC), 77.61 ± 7.71 (NTC)), respectively. Similarly, the maximum tensile stress was demonstrated among group C specimens (Relyx ARC: 69.89 ± 4.81 (NTC), 64.06 ± 4.36 (TC)) with the least in group A (zinc phosphate: 43.66 ± 5.02 (NTC), 37.70 ± 5.10 (TC)]. Table 2 presents means and standard deviation of the maximum load and tensile bond strength of zirconia posts among the study groups.

Furthermore, analyzing the impact of thermocycling that mimics the oral cavity condition pointed out that a significant change in the tensile strength and the maximum load was observed. Among all cements, RMGIC (Maximum load difference: 18.17, Tensile strength difference: 5.23) showed the least impact on bond strength after thermocycling with respect to maximum load followed by RC (Maximum load difference: 24.74, Tensile strength difference: 5.83) and ZPC (Maximum load difference: 33.96, Tensile strength difference: 5.96). 

Comparing the failure modes of study groups, adhesive failures were observed in 100% of specimens in group A (ZPC), 80% in group C (RBC) and 70% in group B (RMGIC), for NTC samples (Table 3). However, among TC specimens, 100%, 90% and 90% adhesive failures were observed in groups A, B and C samples, respectively.

## 4. Discussion 

The present study evaluates the pull out tensile bond strength of a customized ZrO post cemented with ZPC, RMGIC and RC cements in endodontically treated premolars. A significant difference among the cement groups of ZPC, RMGIC and RC was observed, indicating the significance of the cement composition and setting process for bond strength to the ZrO post. Polymeric dual cure cement displayed the highest resistance to retention among all the luting agents for the ZrO post. The rationale for these findings is manifold, including the cementation technique, cement composition and interaction between dentin and luting cements.

Studies have revealed that the apical area of the dentinal root is less organized and more irregular than the coronal portion, contributing to limited pull out bond strength at apical canal regions with a compromised hybrid layer [16]. In addition, operators have increased the irrigation duration for the apical canal to minimize a poor dentin interaction; however, no evident outcomes were perceived [17,18]. Moreover, increasing preparation depth for enhancing the post retention did not influence the retention in the deeper portion and increased the likelihood of root fractures [19]. In the present study, single rooted first premolar teeth were used to manage the root lengths and number of canals. In addition, anterior maxillary incisors are difficult to obtain; by contrast, first premolars are commonly extracted for orthodontic treatments. Therefore, the present study utilized pull out tensile bond strength in premolars in contrast to a sectional technique for bond strength assessment, which avoids the irregular bond strength outcomes in the apical specimens and allows for a bond strength assessment in an orally simulated technique. The present study compared three cements based on their mechanism of adhesion, which included ZPC (friction mechanism), RMGIC (chemical bonding) and RC (micro mechanical). The study showed comparatively better outcomes with polymeric RC cement, which has the capacity to penetrate the dentinal tubules, demonstrate self activation of polymerization and minimize the risk of over etching or over drying in contrast to frictional retention and chemical adhesion [8]. 

Studies suggest that the strength of the cement is not directly related to the retention it provides for the dental root post [20,21]. In addition, evidence demonstrates that friction plays a vital role in providing post retention [22]. Investigations to enhance post retention by covering the post with an extra coat of cement failed to indicate that intimate contact reduces adhesion between the post, cement and root dentin [23,24]. In addition, adhesive bonding to root dentin with high affinity functional resin monomers in a thin layer of polymeric cement has shown improved bond longevity for the luting post [25]. Findings of the present study showed that the friction mechanism for zinc phosphate (non-polymeric) showed the least capability to resist tensile stresses with the least tensile strength compared to conventional polymeric resin cement with an active bonding ability to root dentin showing maximum tensile strength. It is pertinent to mention that non polymeric zinc phosphate cements provide relatively higher fracture resistance as a luting cement; however, luting posts with polymeric cements result in more restorable failures [20].

Conventional polymeric resin luting agents are preferred for cementation of the zirconia post to achieve adequate cement polymerization along the entire root canal space and to obtain an initial stabilization of the post [26,27]. This is in line with the findings of the present study. However, the chemical conversion of monomers to polymers is slow; hence, the light passing through the ceramic post decreases with the depth of the post, compromising the degree of monomer conversion and strength of cement [27]. In addition, studies recommend dual cure polymeric resin cements as they allow for chemical chelation between functional acid methacrylate and calcium from dentinal tissues; however, for longer roots, the use of non polymeric zinc phosphate or hybrid polymer RMGIC is also indicated [17,28]. This is supported by the fact that phosphoric acid within zinc phosphate cement increases the roughness and wettability of apical root dentin, allowing for improved micromechanical retention and bonding [27]. Modified polymer RMGIC on the other hand produces chemical adhesion of a mechanically strong hybrid resin and micromechanical bonding due to the formation of resin tags with the smear layer and root dentin [29].

Studies have employed thermocycling with 40,000 cycles or more to mimic the oral environment in order to understand its dynamics in the in-vitro environment on material performance in the oral cavity [30]. The cyclical stress may cause any debonded regions at the interfaces to grow progressively in size [31]. Similarly, in the present study, it was evident that the thermocycled specimen was associated with lower resistance to withstand maximum load and tensile stress compared to the NTC group specimen. Previous studies show a significant decrease in mean bond strength values observed for the specimen exposed to repeated thermal cycles [31]. However, in the study by Mazzitelli et al., exposure to thermal cycles did not show a significant loss in push-out bond strength for self-adhesive hybrid polymer cements [32]. This suggests that the type, composition and bonding mechanism of luting agents influence the adhesive bonding of the posts to root dentine.

The selection of the recommended cementation technique is key to lower the risk of post and core failure [33]. Studies show that cementation using polymeric resin-based cement includes formation of the hybrid layer and resin tags along with chemical bonding and micromechanical retention. On the other hand, adhesion using ZPC and hybrid polymer RMGIC mainly depends upon the frictional mechanism and chemical stabilization, respectively [34,35]. Authors have described that classifying these bond types is essential to determine the strength of the bond and post stability [36]. The present study showed that the chemical bond is much stabler than physical frictional retention, as it is stable against hydrolysis in the oral cavity. In addition, the chemical interactions are augmented with deeper resin tags, which proceed to thicken under moisture and thereby improve bond strength. This is a possible explanation for the better performance of conventional polymeric RC (RelyX ARC) compared to ZPC and RMGIC. A critical aspect in luting posts is the remaining tooth structure and dentin substrate. Although limited studies have performed pull out bond strength assessments on posts, in a study by Rezaei-Soufi et al. [37], higher pull out bond strength values in premolar teeth were observed in contrast to the present study. A major reason for these findings is the use of a fiber post system in their experiment. Fiber posts are a composition of resin composite modified fibers, which inherently adhere to the resin cements, therefore providing an increased adhesive bond to resin cements [37]. It is difficult to comment on the comparative bond strengths of the customized zirconia post and the fiber post based on the results of the present study, as fiber posts were not used. Therefore, further studies comparing customized fiber and Zr posts with different luting cements using the pull out methodology are recommended.

Within the limitations of the study, a significant difference in the pull out strength of the ZrO post under simulated oral conditions with thermocycling was observed. It is pertinent to mention that the posts used were customized, and the materials used were applied under ideal in-vitro conditions; therefore, outcomes should be applicable to only the materials investigated. In addition, the oral environment is dynamic with exposure to repeated short lived but high occlusal loads on post restored teeth. The present study assessed static loads, which have low failure predictive power, and clinical failures manifest under cyclic subcritical loads. A recent study by Serino et al. reported the effect of cyclic loading and fatigue testing with cement curing duration on dual cured resin cements, showing a significant influence of a 120 s curing time on cement mechanical enhancement [38]. Moreover, factors related to tooth preparation, cement thickness, remaining coronal dentin, preparation of retentive features and tooth type influence post retention in endodontically treated teeth [35,36,39]. As the present investigation was performed in premolars, with a constant preparation design of the root canal, further studies are recommended to explore the influence of polymeric resin cementation techniques and corresponding tooth related factors on the adhesive bond strength of the ZrO customized post in different posterior teeth. 

## 5. Conclusions

Conventional polymeric resin based cement demonstrated a higher bond strength and efficient adhesive bonding of the customized ZrO post with root dentin compared to zinc phosphate (non-polymeric) and RMGIC (hybrid polymer). Thermocycling compromised ZrO post adhesive bonding to root dentin. Chemical bonding with a hybrid layer formation with resin tags by polymer cement enhanced the pull out strength of customized ZrO posts compared to physical frictional retention offered by Zinc phosphate (non-polymeric) and RMGIC (hybrid polymer).

## Figures and Tables

**Table 1:** Cementing protocol for material used in the study protocol.

Cement Type	Manufacturer	Cementing Protocol
Zinc phosphate cement (ZPC)	DeTrey Dentsply Sirona, Charlotte, NC, USA	Gently dry canal preparation with paper points and avoid desiccation. Dispense powder and liquid on a pad, mixing 2.8 to 1 g, (P/L ratio) for 1.30 min; add powder in increments. Apply the creamy mix to the post surface from the apex to the coronal part and insert the post gently to the required length. Remove excess cement.
Resin modified glass ionomer cement (RMGIC)	GC Fuji PLUS Capsule, GC America, Alsip, IL, USA	Apply GC Fuji PLUS conditioner on the prepared canal surface for 20 s for optimal adhesion. Rinse thoroughly with water. Remove excess moisture by paper points or gently blowing with an oil-free air. Dentin should appear moist (glistening). Mix equal parts of powder rapidly with liquid for 20 s (total 30–40 s). Cover the post with cement and insert post gently to required length. Remove excess cement.
Polymeric resin-based cement (RC)	RelyX™ ARC, 3M ESPE, MN, USA	Root canal dentin was etched with phosphoric acid for 10 s and washed and dried with oil free air and paper points. Scotchbond adhesive was applied with microbrush and scrubbed against the dentin surface, and excess was removed and light cured for 10 s. Cement was dispensed to form the 3 M clicker and mixed for 10 s. The post was coated with the cement and gently placed in the canal to full length. Excess was removed at 3 min and photo-polymerized (40 s).

**Table 2:** Means and standard deviation of maximum load and tensile strength of zirconia posts.

Study Groups	Maximum Load Mean (SD)		Tensile Bond Strength Mean (SD)	
Cement Type	NTC	TC	NTC	TC
(A) Zinc phosphate	77.16 (7.71) ^Aa^	43.20 (3.11) ^Ab^	43.66 (5.02) ^Aa^	37.70 (5.10) ^Ab^
(B) RMGIC	105.77 (6.24) ^Ba^	87.60 (6.38) ^Bb^	59.85 (3.53) ^Ba^	54.62 (5.73) ^Bb^
(C) Rely X ARC	123.51 (9.81) ^Ca^	98.77 (8.50) ^Cb^	69.89 (4.81) ^Ca^	64.06 (4.36) ^Cb^
p value	0.003	0.020	0.031	0.026

NTC: no thermocycling, TC: Thermocycling. Dissimilar superscript capital alphabet in same column denotes statistical difference. Dissimilar superscript small alphabet in same row for load or stress denotes statistical difference.

**Table 3:** Failure modes among the tested study groups.

	NTC			TC		
Cement Type	Adhesive	Cohesive	Mixed	Adhesive	Cohesive	Mixed
(A) Zinc phosphate	100	0	0	100	0	0
(B) RMGIC	70	10	20	90	10	0
(C) Rely X ARC	80	0	20	90	10	0

## Data Availability

The data are available on contact from the corresponding author.
